# Carbon dioxide uptake in a eutrophic stratified reservoir: Freshwater carbon sequestration potential

**DOI:** 10.1016/j.heliyon.2023.e20322

**Published:** 2023-09-20

**Authors:** Jinichi Sakaguchi, Keisuke Nakayama, Katsuaki Komai, Atsushi Kubo, Taketoshi Shimizu, Junpei Omori, Kohji Uno, Tomoyasu Fujii

**Affiliations:** a Graduate School of Engineering, Kobe University, 1-1 Rokkodai-Cho Nada-Ku, Kobe City, 658-8501, Japan; b School of Earth, Energy and Environmental Engineering, Kitami Institute of Technology, 165 Koen-cho, Kitami, 090-8507, Japan; c Department of Geoscience, Shizuoka University, 836 Ohya, Suruga-Ku, Shizuoka, 422-8529, Japan; d Water Quality Laboratory, Kobe City Waterworks Bureau, Kobe, Hyogo, 652-0004, Japan; e Department of Civil Engineering, Kobe City College of Technology, 8-3 Gakuenhigahimachi, Nishi-ku, Kobe City, 651-2194, Japan; f School of Science Education, Nara University of Education, Takabatake-Cho, Nara, 630-8528, Japan

**Keywords:** pCO_2_, Stratification, Net ecosystem production, Algae, Freshwater lake

## Abstract

Carbon capture and storage due to photosynthesis activities has been proposed as a carbon sink to mitigate climate change. To enhance such mitigation, previous studies have shown that freshwater lakes should be included in the carbon sink, since they may capture as much carbon as coastal areas. In eutrophic freshwater lakes, there is uncertainty about whether the equilibrium equation can estimate the partial pressure of carbon dioxide (pCO_2_), owing to the presence of photosynthesis due to phytoplankton, and pH measurement error in freshwater fluid. Thus, this study investigated the applicability of the equilibrium equation and revealed the need to modify it. The modified equilibrium equation was successfully applied to reproduce pCO_2_ based on total alkalinity and pH through field observations. In addition, pCO_2_ at the water surface was lower than the atmospheric partial pressure of carbon dioxide due to photosynthesis by phytoplankton during strong stratification. The stratification effect on low pCO_2_ was verified by using the Net Ecosystem Production (NEP) model, and a submerged freshwater plants such as *Potamogeton malaianus* were found to have high potential for dissolved inorganic carbon (DIC) sequestration in a freshwater lake. These results should provide a starting point toward more sophisticated methods to investigate the effect of freshwater carbon on DIC uptake in freshwater stratified eutrophic lakes.

## Highlights

• Intensifying stratification enhances the reduction of pCO_2_ at the water surface.
• Carbon flux from water surface to air can be predicted with the Wedderburn number.
• Residence time longer than 50 days may reduce carbon dioxide absorption.
• Phytoplankton has a high potential for carbon dioxide sequestration.

## Graphical abstract

**Figure:**
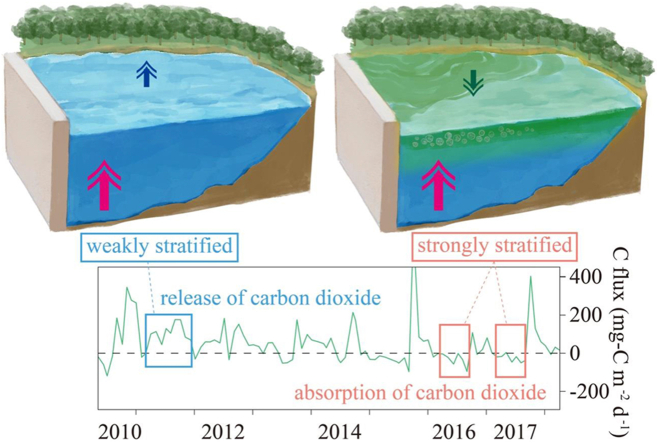

## 1 Introduction

Natural disasters, such as flood inundations, landslides, forest fires, and drought, have occurred worldwide due to climate change, underscoring the urgent necessity of measures to mitigate global warming [1,2]. While various adaptation measures have been applied to climate change, Nellemann (2009), [3]; revealed that blue carbon ecosystems based on submerged aquatic vegetation (SAV) are responsible for capturing and storing approximately 55% of the total CO_2_ sequestered by photosynthesis. Such high sequestration in blue carbon ecosystems is mainly attributable to the high biodiversity existing in coastal regions due to the mixing of fresh and oceanic waters, such as those from estuaries and lagoons. Another contributory factor is that the hydraulic retention (residence time) is longer in coastal regions than in other areas because of the highly closed nature of such regions [[4], [5], [6], [7]].

On the other hand, freshwater lakes are also considered highly closed systems. The total surface water area of estuaries is about 1.8 million km^2^ [3], but the total lake surface water area is more than twice that, at about 5.0 million km^2^ [8,9]. Thus, compared to coastal regions, freshwater lakes may have comparable or greater surface area with potential application to such as a teal carbon ecosystem [10,11]. CO_2_ in freshwater lakes is generally oversaturated, and releases from the lake water surface into the atmosphere [12]. However, Lin et al. (2022), [13]; found that phytoplankton in a subtropical shallow mountainous lake took up and stored CO_2_ through photosynthesis [14]. They further showed that stratification inhibits the vertical flux of the partial pressure of carbon dioxide (pCO_2_) from the lower to the upper layer. This inhibition enhances low pCO_2_ at the water surface due to the photosynthesis effect even though accumulated particulate organic matter releases high concentration of dissolved inorganic carbon (DIC) from the lake bottom.

Among water temperature, salinity, total alkalinity (TA), and DIC, TA has been considered the most constant variable when there is no calcification [15,16]. Therefore, there is a possibility of verifying the effectiveness of freshwater sequestration of carbon by clarifying DIC variations, including inflow and outflow [17,18]. Lin et al. (2021, 2022) [13,14]; investigated DIC flux using a conceptual DIC model, enabling the estimation of net ecosystem production (NEP: the DIC difference between lake and inflow) in a freshwater lake. Theoretically, NEP is a function of residence time and the DIC difference between the lake water and inflow. NEP can be evaluated by DIC uptake by phytoplankton and SAV and DIC flux from lake bottom sediment, meaning NEP can be used to estimate CO_2_ uptake and release when positive and negative, respectively. However, the previous studies focused on vertically well-mixed water bodies [7]; the stratification effect on pCO_2_ has not been studied sufficiently in a eutrophic lake. Also, a very eutrophic condition is associated with a long residence time, which may affect pCO_2_.

Therefore, this study aims to investigate the stratification and hydraulic retention effects on pCO_2_ in a eutrophic lake with stratification. The target lake is Karasuhara Reservoir. Because this reservoir has no SAV it is possible to study the effect of exclusively phytoplankton on pCO_2_. In the Karasuhara Reservoir, TA and pH have been measured for over ten years in order to supply drinking water with appropriate pH, making this a suitable body of water for investigating long-term variation of pCO_2_ in a freshwater lake. However, in a eutrophic lake with high levels of phytoplankton, there is a possibility of significant uncertainty in the estimation of pCO_2_ using TA and pH because of in-situ measurement errors, the presence of organic acid [19], and pH measurement error in freshwater fluid [20]. Thus, we proposed a new equilibrium equation for estimating pCO_2_ using TA and pH with field observations of phytoplankton and dissolved oxygen. In addition, we investigated the stratification effect on pCO_2_ using field observations from 2010 to 2018 in the Karasuhara Reservoir. Finally, we investigated freshwater carbon sequestration potential in a stratified eutrophic lake in terms of CO_2_, such as the DIC difference between the lake and inflow, using a simple Net Ecosystem Production (NEP) model.

## 2 Materials

### 2.1 Field observations

**Fig. 1:**
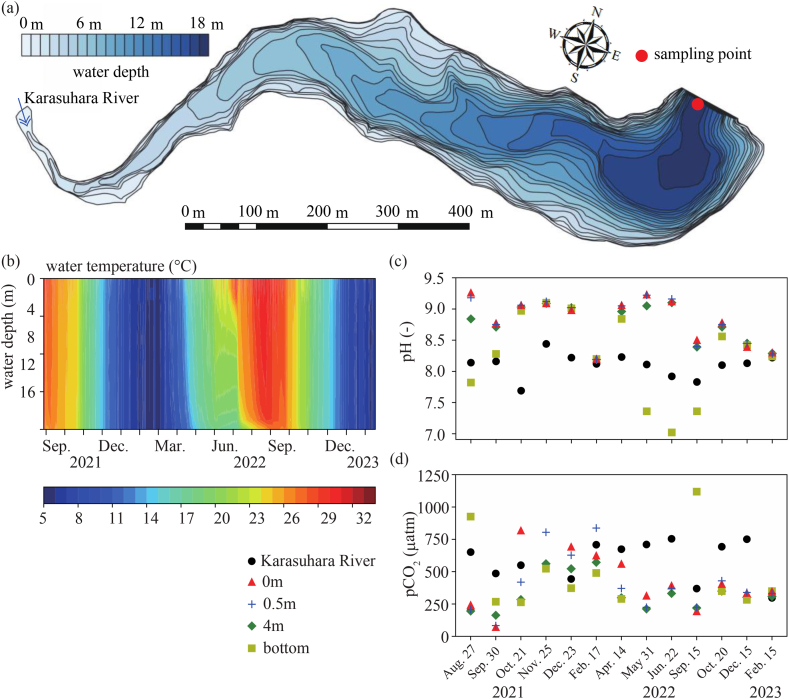
Field observations of the Karasuhara Reservoir from August 2021 to February 2023. (a) Water depth of the Karasuhara Reservoir．The red circle indicates a field observation station. (b) Vertical profile of the water temperature. (c) pH at the water surface, 0.5 m, 4 m, lake bottom, and river. (d) pCO_2_ at the water surface, 0.5 m, 4 m, lake bottom, and river.

The Karasuhara Reservoir is a freshwater lake located at N34°41′30″ and E135°9′19″ in Kobe City, Hyogo Prefecture, Japan. The water surface area is 115,396 m^2^, the total volume is 1,154,000 m^3^, and the maximum water depth is 19 m, with an inflow from the Karasuhara River (Fig. 1a). Aeration devices were installed in the Karasuhara Reservoir and operated from May to October 2021, resulting in weak stratification even during summer (Fig. 1b). Aeration devices are used to pump air into the lower layers of water to improve its quality. The devices help to increase the oxygen levels in the deeper parts of the Karasuhara Reservoir, where oxygen is depleted due to the lack of circulation and decomposition of organic matter at the accumulated bottom sediment. Also, the devices enhance vertical mixing, resulting in a weaker stratification in the reservoir. The bottom aeration system comprises an air compressor, diffusers, and tubing. The compressor pumps air through the tubing and into the diffusers, which release the air into the water at the bottom of the reservoir. The released air rises to the surface, creating a water flow that helps circulate the entire reservoir. The mean values of nitrate nitrogen, ammonium nitrogen, total phosphorus and chlorophyll *a* (chl. a) at the water surface were about 0.116 mg L^−1^, 0.0122 mg L^−1^, 0.0284 mg L^−1^ and 20.5 μg L^−1^ from 2010 to 2018 based on the monthly measured data. There is only one inflow and the discharge is controlled to be constant at 0.24 m^3^ s^−1^, even during the flood period, which provides a residence time of 56 d. Therefore, the Karasuhara Reservoir is considered a typical eutrophic freshwater lake. Field observations were conducted every month from the viewpoint of water quality management to propose a new equilibrium equation for estimating pCO_2_ using TA and pH. We measured water temperature, TA, pH, pCO_2_, and DIC using water samples at 0 m, 0.5 m, 4 m, and the reservoir bottom from August 2021 to February 2023. pH and pCO_2_ were measured using the glass electrode method (LAQUA F-73; Horiba) and the septal electrode method (CGP-31; TOA-DKK). DIC and TA were measured using a total alkalinity titrator (ATT-15; Kimoto Electric). A thermistor chain was deployed to measure the vertical profile of the water temperature with a vertical interval of 1 m from August 2021 to February 2023 (Onset U22-001 Water Temperature Pro v2 Data Logger; HOBO).

In addition, we measured water temperature, TA, and pH using water samples at 0 m, 0.5 m, 4 m, and the lake bottom from 2010 to 2018. Since we measured the long-term vertical profiles of water temperature, pH, and TA, enabling us to estimate pCO_2_, we investigated the effect of stratification on pCO_2_ by dividing the year into four seasons. Generally, stratification becomes stronger from spring to autumn compared to winter, excluding the typhoon period. The measurement accuracies of DIC and TA are about ±5 μmol kg^−1^ and ±0.001 for pH. Therefore, the possible precision error is ±36 μatm of pCO_2_, which is estimated by using Zeebe et a. (2001) [21]. In the analysis, The Brunt–Väisälä frequency was calculated using the following equation (1):

(1) $$N_{B}^{2}=-\frac{g}{\rho }\frac{\partial \rho }{\partial z}\approx \frac{\varepsilon g}{H}$$

where *N*_*B*_ (s^−1^) is the Brunt–Väisälä frequency, *ρ* (kg m^−3^) is the water density, *z* (m) is the vertical coordinate, *g* (m s^−2^) is the gravitational acceleration, ε is the specific density difference, and *H* (m) is the mean total depth (=10 m).

### 2.2 Modification of chemical equilibrium equations

The direct measurement of pCO_2_ is the best method to investigate and analyze carbon flux (C flux) from the water surface to the atmosphere, especially at freshwater sites (e.g., Refs. [22,23]). Unfortunately, pCO_2_ in many lakes is not measured directly in Japan. Nonetheless, TA and pH have been measured in many lakes for over ten years, particularly in reservoirs serving as drinking water. Thus, many previous studies have attempted to estimate pCO_2_ using an equilibrium equation with two of the three parameters TA, DIC, and pH, rather than by direct measurement of pCO_2_ based on the detection principle of a nondispersive infrared sensor (NDIR) [24,25]. However, the equilibrium equation overestimates pCO_2_ due to TA with low carbonate alkalinity and high DOC concentrations in acidic and organic-rich waters [23]. In contrast, the equilibrium equation has been verified using a water sample with a pH of less than 8.4, as shown in Cai & Wang (1998) [24];, Dickson (1990) [26];, Dickson et al. (2007) [27]; and Moore-Maley et al. (2016) [28]. Since the Karasuhara Reservoir is a typical eutrophic reservoir with extensive phytoplankton, the pH is usually more than about 8.0 in the upper layer. Thus, we modified the equilibrium equation shown by Zeebe et al. (2001) [21]; and investigated the applicability of the new equilibrium equation, which modifies pH values only. Note that we confirmed the use of the equilibrium equation by Millero et al. (2010) [29]; for lower salinity: it gave values almost equivalent to those of Zeebe et al. (2001) [21].

### 2.3 Net ecosystem production model to estimate the effect of stratification on C flux

We attempted to develop a simple Net Ecosystem Production (NEP) model to understand the effect of stratification on C flux from the water surface to the atmosphere. Photosynthesis is one of the dominant factors controlling DIC in association with dissolved oxygen (DO) in the Karasuhara Reservoir (see the Discussion). Therefore, the photosynthesis effect was modelled using a function of DO. The NEP model also included DIC inflow, C flux from the water surface to the atmosphere, and DIC flux from the bottom or the lower layer to the effective-volume layer. The effective-volume corresponds to an upper layer when stratification is formed clearly; otherwise, the effective volume equals the entire volume. The detailed relationship is expressed in the following equation:

(2) $$C_{0}\frac{\partial DIC_{S}}{\partial t}=-\alpha_{U}\left(\frac{DO}{100}-\beta_{U}\right)+C_{0}\left(DIC_{R}-DIC_{S}\right)\frac{Q_{R}}{V_{E}}-\frac{A_{S}}{V_{E}}F_{DIC}+\frac{A_{B}}{V_{E}}B_{DIC}$$

wher $e\;V_{E}$ is the effective volume (m^3^), $DIC_{S}$ is the DIC (μmol kg^−1^) in a reservoir, $\alpha_{U}$ is the uptake coefficient of CO_2_ by phytoplankton (mg-C m^−3^ d^−1^), $\beta_{U}$ is the parameter for phytoplankton photosynthesis, $C_{0}$ is the coefficient from μmol kg^−1^ to mg-C m^−3^, DO is the DO concentration (%) in a lake, $Q_{R}$ is the inflow (m^3^ s^−1^), $DIC_{R}$ is the river DIC (μmol kg^−1^), $A_{S}$ is the water surface area (m^2^), $F_{DIC}$ is the C flux from the water surface to the atmosphere (mg-C m^−2^ d^−1^), $A_{B}$ is the lake bottom area (m^2^), and $B_{DIC}$ is the DIC flux from the bottom (mg-C m^−2^ d^−1^).

We applied the Wedderburn number [30], $W_{N}$, to include the stratification effect on the effective volume and the DIC flux from the bottom or the lower layer to the effective-volume layer by introducing the criterion of the Wedderburn number, $W_{NC}$. When the Wedderburn number is less than $W_{NC}$, the upwelling becomes dominant, with the result that the effective volume equals the entire volume of the Karasuhara Reservoir with a mean total depth of 10 m. In contrast, when the Wedderburn number is more than $W_{NC}$, the effective volume equals the upper layer with a water depth of 5 m due to the suppression of vertical mass flux by stratification as equations (3), (4):

(3) $$W_{N}=\frac{\varepsilon gh^{2}}{u_{*}^{2}L}=\frac{N_{B}^{2}hH}{u_{*}^{2}}\frac{h}{L}=Ri\frac{h}{L}$$

(4) $$\left\{ \begin{array}{ccc} \text{stratified}:W_{N}>W_{NC} & h=5\;m & B_{DIC}=B_{S}\\ \text{turnover}:W_{N}\leq W_{NC} & h=H=10\;m & B_{DIC}=B_{L} \end{array}\right.$$

where $u_{*}^{2}$ (m^2^ s^−2^) is the friction velocity at the water surface due to wind, L (m) is the representative length of a reservoir (=650 m), and Ri is the Richardson number ($=N_{B}^{2}hH/u_{*}^{2}$).

In equation (2), the unknown parameters are $\alpha_{U}$, $\beta_{U}$, and $B_{DIC}$. Initially, because we only considered the effect of phytoplankton photosynthesis when DO exceeds 100%, $\beta_{U}$ was set to 1.0. Therefore, when DO was less than or equal to 100%, $\alpha_{U}$ was set to 0. Next, we varied $\alpha_{U}$ (mg-C m^−3^ d^−1^) and $B_{DIC}$ (mg-C m^−2^ d^−1^) between 0 and 2000 to calculate the most appropriate DIC values. We took the difference between the estimated and observed DIC for the target month and found the most fitting parameter values that minimized the estimation error.

## 3 Results

### 3.1 Field observations and laboratory experiment

The water temperature difference between the upper and lower layers was about 4° in August 2021 and decreased slightly in September 2021 (Fig. 1b). There was no water temperature difference between the water surface and the reservoir bottom from October 2021 to April 2022. Strong stratification was formed from May to September in 2022. pCO_2_ at the water surface was lower than the atmospheric pCO_2_, 440 μatm, in August and September in 2021 and May to September in 2022 due to the photosynthesis effect of phytoplankton (Fig. 1d). Below, the photosynthesis effect on pCO_2_ will be discussed in terms of the oxygen demand and pCO_2_ (see the Discussion). In contrast to the pCO_2_ at the water surface, the pCO_2_ adjacent to the reservoir bottom was greater when stratification was strong, such as in August 2021 and September 2022. The minimum and maximum pH values were 7.7 and 9.5 (Fig. 1c). The maximum pH occurred at the water surface during strong stratification.

### 3.2 Modification of the chemical equilibrium equations

**Fig. 2:**
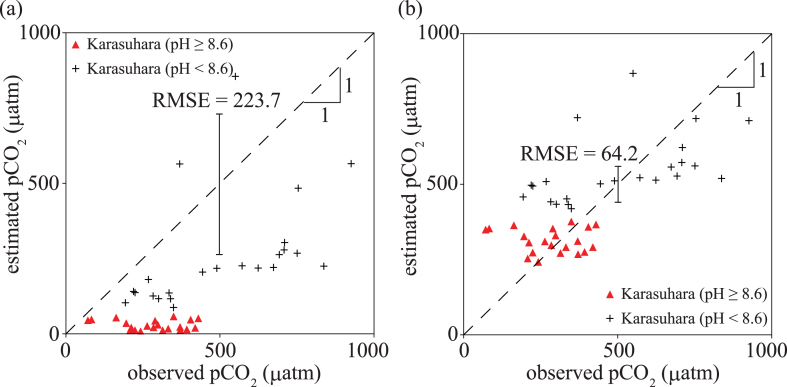
Observed and estimated pCO_2_. (a) pCO_2_ estimated from water temperature, pH and TA using the original equilibrium equation. (b) pCO_2_ estimated using the proposed pH correction equation.

The estimated pCO_2_ values obtained by the equilibrium equation [21] using water temperature, pH and TA from the field observations disagreed with the directly measured pCO_2_ values (r: 0.63; p-value: 0.000005; root mean square error (RMSE): 223.7) (Fig. 2a). The correlation coefficient was relatively high because linear regression has a high correlation between the observed and estimated pCO_2_. However, most importantly, the estimated pCO_2_ values underestimated the directly measured pCO_2_ values when pH was in a range of 7.7–9.5 from the field observations. In particular, when pH was over 8.6, the estimated pCO_2_ was much smaller than the directly measured pCO_2_, suggesting that the larger the pH, the less the estimated pCO_2_ agrees with the actual values.

Therefore, we corrected the pH values using the actual water temperature, TA and pCO_2_ in an attempt to improve the accuracy of pH measurement as equation (5):

(5) $${\text{pH}}_{\text{cor}}=\left\{ \begin{array}{cc} {\text{pH}}_{\text{obs}} & \text{pH}\leq 7.7\\ \beta {\left[{\text{pH}}_{\text{obs}}\right]}^{\gamma } & \text{pH}>7.7 \end{array}\right.$$

where ${\text{pH}}_{\text{cor}}$ (−) is the corrected pH for the equilibrium equation, β and γ are the parameters for the pH correction equation, and ${\text{pH}}_{\text{obs}}$ (−) is the observed pH.

**Fig. 3:**
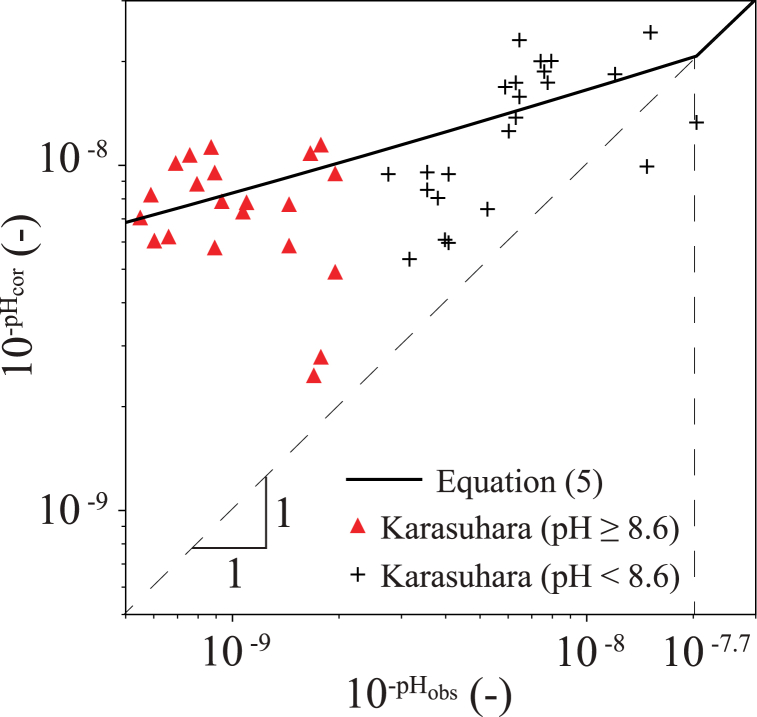
Observed pH and corrected pH. The coefficients β and γ are 4.0 and 0.32.

The correction coefficients for the pH correction equation were obtained from the comparisons with the field observations (Fig. 3). The corrected and observed pH values were significantly well represented using a linear regression line, with an r^2^ of 0.58 and a p-value of 0.00004. The larger the pH, the larger the correction needed. A pH less than 7.7 was the criterion for applying pH correction. As expected, a more substantial correction was found to be needed for a larger pH (pH > 8.6). We compared the estimated pCO_2_ through the equilibrium equation using the corrected pH from the field observations (Fig. 2b). The estimated pCO_2_ agreed well with the directly measured pCO_2_, with an r^2^ of 0.65 and a p-value of 0.000002 and an RMSE of 64.2, suggesting that the modified pH should be used rather than the observed one. As the possible precision error is ±36 μatm of pCO_2_ in this study, we note that the corrected pCO_2_ has error larger than the measurement error using Zeebe et al. (2001) [21].

### 3.3 Influence of stratification on pCO_2_

**Fig. 4:**
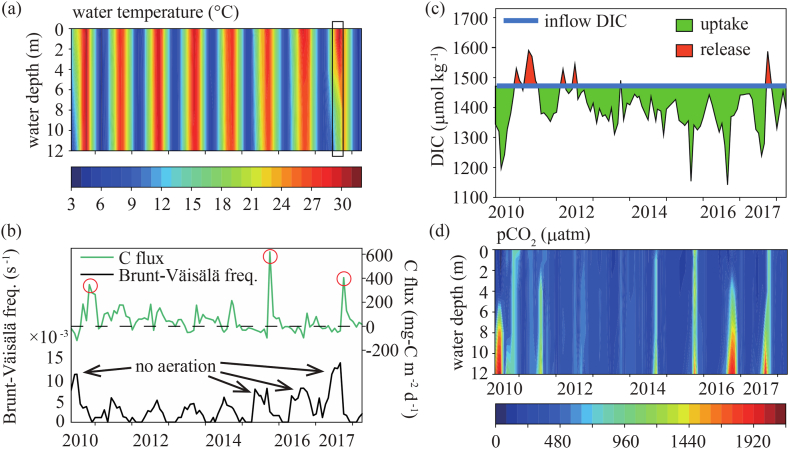
Field observations at the Karasuhara Reservoir from 2010 to 2018. (a) Vertical profile of water temperature. (b) Brunt-Väisälä frequency and carbon flux from the water surface to the atmosphere. Red circles indicate the sudden increase in DIC due to the turnover. (c) DIC at the water surface. (d) Vertical profile of pCO_2_.

The Brunt–Väisälä frequency in the Karasuhara Reservoir was higher in 2010, 2015, 2016, and 2017, when the aeration device was not operated, than in the other years (Fig. 4b); the difference was especially great in 2017 (black rectangle in Fig. 4a). The stratification suppressed the vertical DIC flux, resulting in large pCO_2_ in the lower layer because of the DIC release from the lake bottom, where the particulate organic matter had accumulated. In 2017, while the stratification was formed clearly, pCO_2_ was small in the upper layer due to the photosynthesis effect of phytoplankton (Fig. 4d). However, the turnover caused intense vertical mixing, resulting in a more significant C flux from the water surface to the atmosphere (red circles in Fig. 4b). Lin et al. (2021) demonstrated that the difference in DIC between an inside lake and inflow indicates the reduction of DIC due to the uptake by phytoplankton.

**Fig. 5:**
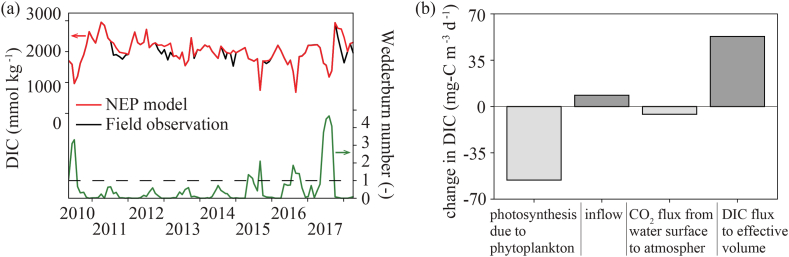
The NEP model results. (a) Wedderburn number and DIC at the water surface. (b) The contributions of each DIC flux to the DIC: photosynthesis of phytoplankton, inflow, C flux from the water surface to the atmosphere, and the DIC flux.

Stevens and Imberger (1996) [31]; showed that upwelling presumably dominates when the Wedderburn number is less than 1.0 ($=W_{NC}$). Thus, the Wedderburn number showed a high possibility of turnover in a reservoir from 2011 to 2014 (Fig. 5a). Therefore, we assume that clear stratification was formed for four years: 2010, 2015, 2016 and 2017. The NEP model demonstrated good agreement with the field observations in the Karasuhara Reservoir, with an r^2^ of 0.99 (Fig. 5a). In addition, the NEP model provided the contribution of each DIC flux component to the DIC in the Karasuhara Reservoir (Fig. 5b). The most significant component was the photosynthesis of phytoplankton, followed by the DIC flux from the bottom or the lower layer to the upper layer. The contribution of inflow and C flux from the water surface to the atmosphere was much smaller than the contribution of the above-mentioned components.

## 4 Discussion

Previous reports have shown that stratification in a subtropical shallow lake inhibits the DIC flux from the lower layer with a high pCO_2_ to the upper layer [13,14]. Our present results also showed that the vertical flux of high pCO_2_ from the lower layer to the upper layer was suppressed, resulting that stratification enhanced the reduction of pCO_2_ in the upper layer compared to the atmospheric pCO_2_ due to photosynthesis by phytoplankton. On the other hand, since vertical mixing breaks stratification in a lake due to radiative cooling during winter, DIC flux in the lower layer reaches the surface quickly, resulting in high pCO_2_. Lin et al. (2021) [13]; revealed that typhoons had a similar effect on vertical mixing, which may mean that the flow field controlled the pCO_2_ profile. Also, it is thought that a decrease in photosynthesis activity caused the increase in pCO_2_ at the water surface during winter. Since the Karasuhara Reservoir is a highly eutrophic lake, primary production of ecosystem due to phytoplankton was high, suggesting DIC values inside the reservoir were smaller than inflow (Fig. 4c).

**Fig. 6:**
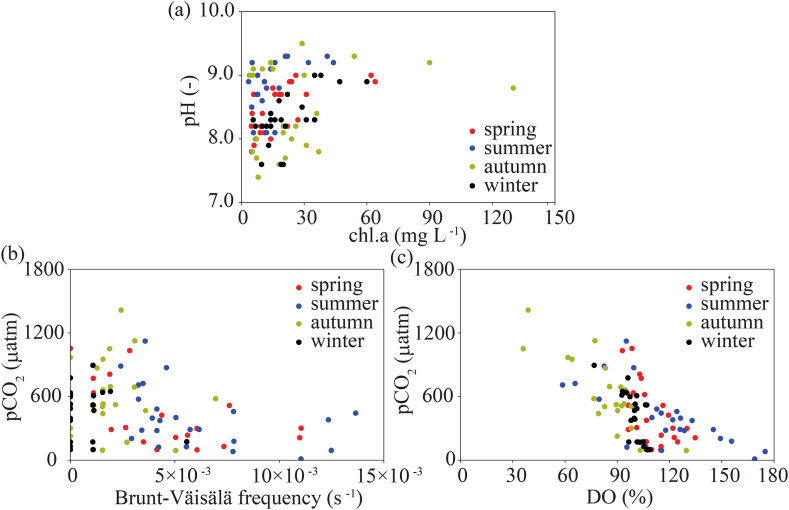
Field observations at the Karasuhara Reservoir from 2010 to 2018. Red, blue, yellow, and black circles show the measurements for spring, summer, autumn, and winter, respectively. (a) Observed chl. a and pH at the water surface. (b) Brunt‒Väisälä frequency and pCO_2_ at the water surface. (c) DO and pCO_2_ at the water surface.

The pCO_2_ estimated by the equilibrium equation using water temperature, TA and pH did not agree with the directly measured pCO_2_ when pH was more than 7.7. The increase in pH above 7.7 was attributed to the increase in phytoplankton (photosynthesis). Previous studies showed that the more phytoplankton there is, the higher the pH in freshwater lakes [[32], [33], [34]]. For purposes of the present analysis, we defined spring as March to May, summer as June to August, autumn as September to November, and winter as December to February. We attempted to compare pH with chl. a using the field observations at the water surface from 2010 to 2018 (Fig. 6a). Although no relationship between pH and chl. a was apparent, there was a direct correlation between chl. a and pH values, particularly in spring. This may suggest that the CO_2_ consumption by phytoplankton causes pH to increase, which would necessitate modification of the pH values. Note that pH was more than 9.0 even though chl. a was lower than 10 μg L^−1^. This suggests that the differences among phytoplankton species should be analyzed in future studies.

Lin et al. (2021, 2022) [13,14]; found that the difference in pCO_2_ between the upper and lower layers in shallow subtropical mountainous lakes became larger due to stratification. Our study also showed the same tendency for pCO_2_ in the upper and lower layers. Thus, we investigated the relationship between pCO_2_ at the water surface and the Brunt–Väisälä frequency, and found that the stronger the stratification, the smaller the pCO_2_ (Fig. 6b). In particular, pCO_2_ at the water surface was suppressed with the increase in the Brunt–Väisälä frequency during summer. Therefore, as pCO_2_ flux from the lower to the upper layer was inhibited, pCO_2_ was decreased due to photosynthesis by phytoplankton. In addition, pCO_2_ at the water surface decreased with the increase in DO from spring to autumn, confirming that the higher the Brunt–Väisälä frequency, the larger the DO (Fig. 6c). Note that low DO during autumn was driven by the collapse of stratification due to turnover, in which low DO is transported to the water surface.

When there is no decrease or increase in DIC due to photosynthesis, DIC flux from the bottom sediment, and C flux at the water surface, DIC in a reservoir becomes equal to inflow DIC in the upper layer. Indeed, DIC decreases from spring to autumn since stratification suppresses the vertical flux from the lower to the upper layer, and photosynthesis becomes active. In contrast, DIC during winter or turnover periods is expected to increase due to the DIC flux from the lower layer (red circles in Fig. 4b) [30]. The other phenomena demonstrated the importance of upwelling on the vertical mixing—a massive methane release from the water surface to the atmosphere—in a brackish lake, suggesting the upwelling may also enhance and release more DIC after collapsing stratification [35]. Therefore, we calculated how much DIC the phytoplankton reduced due to photosynthesis from 2010 to 2018 (Fig. 4c). We obtained a mean inflow DIC of 1470 μmol kg^−1^, yielding a mean DIC in the upper layer of 1366 μmol kg^−1^ for the four years of 2010, 2015, 2016 and 2017 (the strong stratification period) and 1403 μmol kg^−1^ for the four years from 2011 to 2014 (the weak stratification period). The reduction of DIC during the strong stratification was 104 μmol kg^−1^, much greater than that of 67 μmol kg^−1^ during the weak stratification period. This resulted in less C flux from the water surface to the atmosphere (Fig. 4b).

**Fig. 7:**
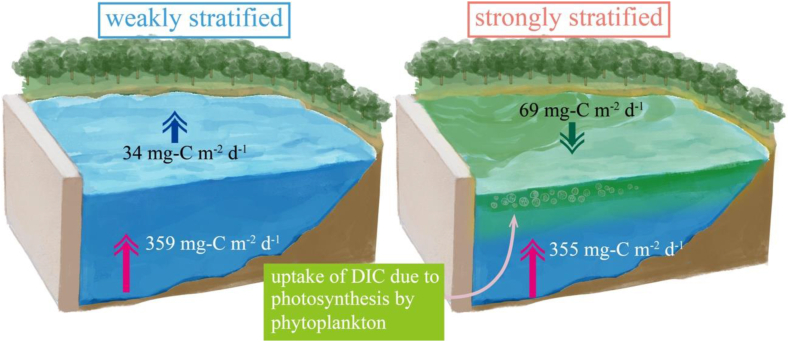
Schematic diagram of carbon flux in the Karasuhara Reservoir. Red arrows show the DIC flux from the lake bottom to the upper layer. Uptake of DIC by phytoplankton is further greatly enhanced, resulting in greater absorption of carbon from the atmosphere to the water surface during the strong stratification period than the weak stratification period. Illustration adapted with permission from Reina Nakayama.

In the NEP model analysis, the total contribution of inflow and C flux from the water surface to the atmosphere corresponds to the NEP in a reservoir; this value was 5.3 mg-C m^−3^ d^−1^. The contribution of photosynthesis due to phytoplankton on NEP was 41.2 mg-C m^−3^ d^−1^, as shown in Fig. 5b. Lin et al. (2021, 2022) [13,14]; demonstrated that the absolute value of the NEP was much larger in shallow subtropical mountainous lakes than in the Karasuhara Reservoir, usually by more than 100 mg-C m^−3^ d^−1^. Since the NEP is inverse to hydraulic retention (residence time), the smaller the inflow, the smaller the NEP. As the inflow to the Karasuhara Reservoir is about 20,700 m^3^ d^−1^, the residence time is about 56 d, much longer than in Lin et al. (2021) and Lin et al. (2022). Therefore, the NEP in the Karasuhara Reservoir was smaller than in the previous studies. Lin et al. (2022) revealed that the residence time needs more than one week for phytoplankton and planktic bacteria to grow and consume DIC due to photosynthesis [[36], [37], [38]]. Therefore, our study revealed that an overly long residence time suppressed the NEP, reducing DIC absorption. Interestingly, the NEP model showed that the DIC flux from the lake bottom to the upper layer was 355 mg-C m^−2^ d^−1^ for the four years of 2010, 2015, 2016 and 2017 (the strong stratification period) and 359 mg-C m^−2^ d^−1^ for the four years from 2011 to 2015 (the weak stratification period). In contrast, when we focus on the influence of stratification using the Wedderburn number, the pCO_2_ flux from the water surface to the atmosphere was −69 mg-C mg-C m^−2^ d^−1^ (DIC absorption from the atmosphere) when $W_{N}>1.0$, and it was 34 mg-C m^−2^ d^−1^ (DIC release from the reservoir) when $W_{N}<1.0$ (Fig. 7). With a stable stratification when $W_{N}>1.0$, water temperature is warmer, and the uptake of DIC by phytoplankton is more further enhanced, resulting in greater absorption of carbon from the atmosphere to the water surface during the strong stratification period than the weak stratification period. Therefore, the phytoplankton activity on DIC due to photosynthesis plays a significant role in the C flux at the water surface in a reservoir.

In such a reservoir with a long residence time, there is a possibility that planted SAVs uptake DIC in the upper layer efficiently and capture CO_2_ from the atmosphere, which may be enhanced by the photosynthesis effect of phytoplankton on the decrease in pCO_2_. Regarding the DIC uptake by SAVs, a DIC equation was proposed by Nakayama et al. (2020a) [6]; to understand the effect of *Zostera Marina* on DIC sequestration in Komuke Lagoon [[39], [40], [41], [42]]. In contrast, Nagatomo et al. (2023) [43]; proposed a DIC equation for *Potamogeton crispus* in Australia's freshwater lake, Lake Monger. They found that *P. crispus* has a higher potential to capture DIC than *Z. marina*. Although it is difficult to directly compare these SAVs since one grows in freshwater and the other in oceanic waters and the vegetation density is greater in Lake Monger than Komuke Lagoon, these findings nevertheless suggest that SAVs in freshwater lakes may exhibit CO_2_ absorption close to that of their coastal counterparts. *Z. marina* is a refractory SAV, indicating that its effect on capturing CO_2_ is as significant as that of blue carbon [3]. The bending effect of *P. crispus* on capturing CO_2_ also has not been revealed. In addition, Nakayama et al. (2020b) [44]; and Matsumura et al. (2022) [45]; demonstrated the importance of deflected vegetation height on DIC absorption. Since *P. crispus* is more elastic than *Z. marina*, the practical DIC absorption volume of the former may be lower than that of the latter. Nevertheless, there is a high possibility that SAVs capture and store CO_2_ from the atmosphere in a freshwater lake bottom.

Collectively, these results reveal three significant aspects of the freshwater carbon ecosystem. First, there is considerable uptake of DIC by phytoplankton even in a weak stratification. Second, stratification enhances the uptake of DIC due to photosynthesis, even though a release of DIC to the effective-volume layer was expected due to turnover in autumn. And third, *P. crispus, which like Potamogeton malaianus is a typical SAV in freshwater lakes, has* high potential for DIC sequestration in a freshwater lake. Although freshwater lakes have been considered to release CO_2_ because of the substantial carbon input from forests, the outcome of this study suggests the importance and benefit of freshwater carbon in lakes, reservoirs, and ponds (like teal carbon in freshwater wetlands) from the viewpoint of DIC [46].

## 5 Conclusion

In a eutrophic freshwater lake where pH usually exceeds 8.0, a comparison between pCO_2_ values calculated by the equilibrium equation and pCO_2_ values measured directly revealed the need to modify the pH values. The proposed equilibrium equation using the corrected pH successfully reproduced the pCO_2_ values measured directly through field observations. We estimated pCO_2_ in the Karasuhara Reservoir from 2010 to 2018 and found that intensifying stratification enhanced the photosynthesis by phytoplankton and the reduction of pCO_2_ at the water surface. The potential photosynthesis effect of freshwater SAVs was discussed and may be close to that of *Z. marina*, a typical coastal SAV. Our outcomes may provide a starting point for further studies to investigate the effect of freshwater carbon on DIC uptake in freshwater stratified lakes.

## Author contribution statement

Jinichi Sakaguchi performed the experiments, and analyzed and interpreted the data; Keisuke Nakayama conceived and designed the experiments, analyzed and interpreted the data, and wrote the paper; Katsuaki Komai performed the experiments; Atsushi Kubo analyzed and interpreted the data; Taketoshi Shimizu performed the experiments, and wrote the paper; Junpei Omori performed the experiments; Kohji Uno performed the experiments; Tomoyasu Fujii performed the experiments.

## Data availability statement

Data will be made available on request.

## Declaration of competing interest

The authors declare that they have no known competing financial interests or personal relationships that could have appeared to influence the work reported in this paper.
